# Novel Validated Index for the Measurement of Disinformation Susceptibility at the County Level

**DOI:** 10.7759/cureus.15305

**Published:** 2021-05-28

**Authors:** Michael X Jin, Sangita Rajan, Carlos E Gary Bicas, Max Hao, Letian Dong, Beckett Mufson, Imran Hafiz

**Affiliations:** 1 Radiology, Stony Brook University Hospital, Stony Brook, USA; 2 Data Science, University of California Los Angeles, Los Angeles, USA; 3 Planetary Scientist, Stony Brook University, Stony Brook, USA; 4 Economics, University of North Carolina, Chapel Hill, USA; 5 Journalism, Hofstra University, Hempstead, USA; 6 Journalism, Duke University, Durham, USA

**Keywords:** disinformation, index, public health, community health, government, corona virus, measurement validation

## Abstract

In the past decade, disinformation has become an increasingly dangerous enemy of public health, scientific advancement, and social stability. To address and counter this trend, it is essential to first identify communities most at risk for disinformation. The Jin-Hafiz Disinformation Index (JHDI) is developed and validated as a tool to counter disinformation and address deficits of good information on a county level in the United States. Once vulnerable communities are identified with the JHDI, targeted interventions with community partnerships can be conducted to address knowledge concerns.

## Introduction

The growth of social media has allowed an increase in viewpoints and voices. However, there is a small but prominent portion of these voices that are not based on facts or, worse, are intentionally misleading. Disinformation, or “false information which is intended to mislead” as defined by Oxford dictionaries, further degrades the trust the public has for institutions that are responsible for producing and verifying information based on facts, including the media, universities, government, and scientific institutions [1]. Mass media and social networks have been fundamental in the management of public health related information in recent years. However, disinformation, misinformation, and fake reports have deluged this avenue of information delivery [2-4]. Disinformation is particularly damaging towards public health and patient care, especially in the midst of the ongoing coronavirus disease 2019 (COVID-19) pandemic.

The COVID-19 pandemic has thrown this epistemic crisis into sharp relief. COVID-19 has killed more than 2 million people and infected nearly 100 million more worldwide. In the United States alone, COVID-19 has killed more than 500,000 people and infected nearly 30 million, which is near a third of the total cases in the world [2]. Yet, despite the staggering mortality and infection rates, nearly 40% of Americans still say they do not intend to take the vaccine. Most frequently cited reasons include uncertainty of vaccine effectiveness and possibility of unknown side effects despite increasing evidence that the vaccine is safe and effective [3]. Tedros Adhanom Ghebreyesus, Director-General of the World Health Organization (WHO), recognized the overabundance of information, misinformation, disinformation, and outdated information causing people to ignore the empirically backed COVID-19 guidelines as an “infodemic” [4].

For those working to disseminate scientifically verified public health information, a primary challenge is discerning and measuring how misinformation impacts a given population's behavior. For the purposes of this paper, we are specifically interested in public health misinformation surrounding COVID-19. We will outline a process for indexing publicly available data to measure the susceptibility of communities in the United States to disinformation and to study the efficacy of anti-disinformation campaigns. This index shall be referred to as “The Jin-Hafiz Disinformation Index” (JHDI) throughout this document.

## Materials and methods

A literature review was conducted to determine demographic factors most associated with disinformation [5-14]. Out of 15 factors, five were ultimately selected for the index after evaluating for availability of data in census and publicly available databases with resolution at a county level, including race, gender, poverty status, education, and unemployment (Table 1). Commonalities in variables were also cross-referenced with the Centers for Disease Control and Prevention (CDC) Social Vulnerability Index (SVI) [15]. These factors were used to create three preliminary indices with different weights placed on each variable in which the sum of each index value is added together to produce the index score (Table 2). Each of the three indices was then validated against disinformation variables from the Yale Climate Survey data [16-18]. The index version that most closely predicted both disinformation variables was selected as the final index.

**Table 1:** Demographic and Social Variables Identified to Have Significant Correlation With Disinformation

Variables selected for index	Variables correlated with disinformation not selected for index due to incomplete of county-level data	Variables correlated with disinformation not selected because political considerations of partisan divide
Population not racial/ethnic minority	Social isolation	Evangelical population
Speaks English less than well	Trust of authorities in science and medicine	
Household below poverty	Physical or mental disability	
Population without high school diploma	Religious belief	
Unemployment	Social influence score	
	Birthplace of parents	
	Population without internet access	
	Property crime rates relative to national mean	
	Higher conservative-leaning population	

**Table 2:** Preliminary Indices RAC = z-score of % population not racial/ethnic minority LANG = z-score of % speaking English less than well POV = z-score of % household below poverty HSD = z-score of % population without high school diploma EMP = z-score of % unemployed

Index Version	Multiplier for RAC	Multiplier for LANG	Multiplier for POV	Multiplier for HSD	Multiplier for EMP
Ver. 1	1x	1x	1x	1x	1x
Ver. 2	2x	1x	1x	2x	2x
Ver. 3	3x	1x	1x	3x	2x

To do this, census data was first filtered based on year (only including the most recent data) and then county codes were converted to Federal Information Processing Standards (FIPS) codes. Columns for population proportions were then created corresponding to:

● Race: people categorized as not racial/ethnic minority

● Speak English less than very well

● Poverty: people below poverty level in the last year

● Educational attainment: people aged 25 years and over who do not have any college degree

● Unemployment: unemployment rate for people aged 20-64 years

County values were calculated for each of the three preliminary indices.

Global warming opinions compiled by the Yale Climate Survey were used to validate the preliminary indices. Four distinct areas from the 63 questions of the survey were selected based on their association with disinformation given the amount of evidence and testable hypotheses presented against such beliefs:

● Estimated percentage who do not think that global warming is happening

● Estimated percentage who think that global warming is caused mostly by natural changes in the environment

● Estimated percentage who believe there is a lot of disagreement among scientists about whether or not global warming is happening

● Estimated percentage who somewhat/strongly disagree that global warming is affecting the weather in the United States

**Figure 1:**
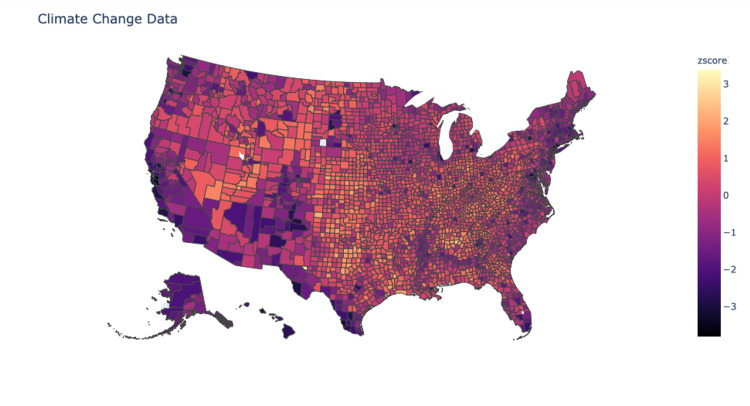
Heat Map for Climate Data

Unweighted means were then calculated for the four variables for each county. Values were normalized and then processed into a heat map of the continental United States (Figure 1). Pearson’s tests were used to compare the correlation of each of the indices with the county values obtained from the Yale Climate Survey.

## Results

**Figure 2:**
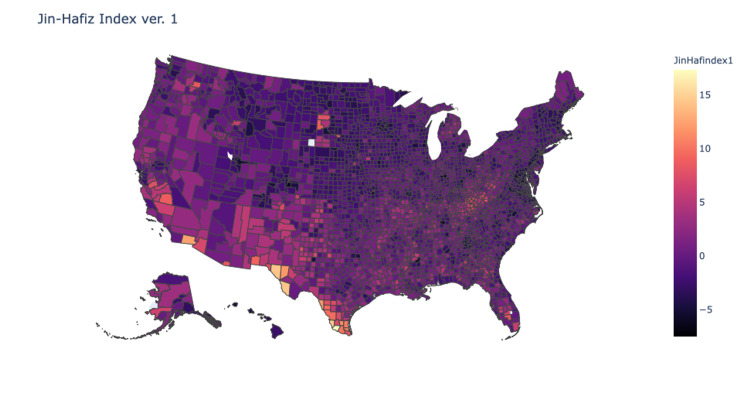
Heat Map of JHDI Version 1 correlation: 0.071; determination: 0.005 JHDI, Jin-Hafiz Disinformation Index

**Figure 3:**
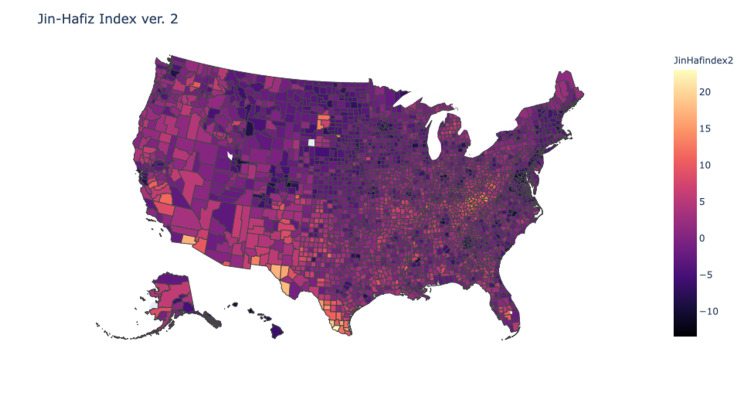
Heat Map of JHDI Version 2 correlation: 0.224; determination: 0.050 JHDI, Jin-Hafiz Disinformation Index

**Figure 4:**
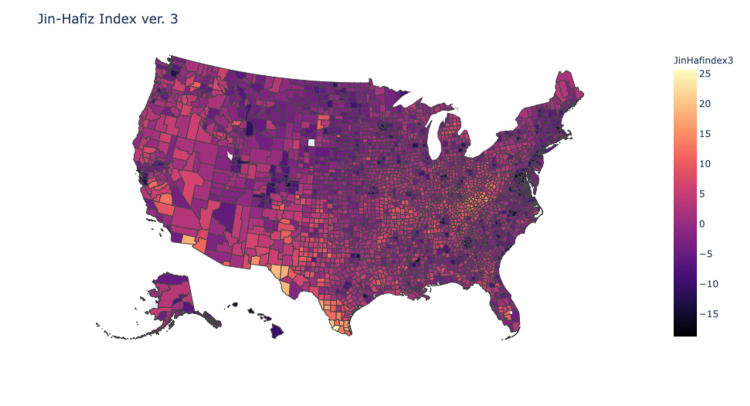
Heat Map of JHDI Version 3 correlation: 0.361; determination: 0.130 JHDI, Jin-Hafiz Disinformation Index

Pearson’s tests were used to compare the correlation of each of the indices with the county values obtained from the Yale Climate Survey. Values for the indices were normalized and then processed into heat maps of the continental United States. Analysis showed that index version 1 demonstrated weak correlation (R=0.1), version 2 demonstrated mild correlation (R=0.2), and version 3 demonstrated moderate correlation (R=0.4) (Figures 2-4). Version 3 of the JHDI demonstrated the best correlation with the Yale Climate Survey in predicting community susceptibility to disinformation (Table 3).

**Table 3:** Final Index Score interpretation: ≤10: Extremely resistant to disinformation -10 to -5:  Above average resistance to disinformation -5 to 5: Average susceptibility to disinformation 5 to 10: Above average susceptibility to disinformation >10: Extremely susceptibility to disinformation

Version 3
z-score of % racial/ethnic minority (x3)
z-score of % speaking English less than well
z-score of % household below poverty
z-score of % population without high school diploma (x3)
z-score of % unemployed (x2)
Total score:

## Discussion

Much of the current discussion regarding disinformation focuses on why and how it spreads. The JHDI seeks to understand the “who” piece of the puzzle: the populations at greatest risk of falling prey to disinformation. In order to fight disinformation, it is essential to determine which communities are most vulnerable to information, especially since falsehoods can spread six times as fast as the truth on social platforms [19]. A recent NPR/Marist poll found that one in four people living in the United States said they would “refuse a COVID-19 vaccine outright if offered” due to largely unfounded fears regarding the vaccine, threatening the prospects of herd immunity in America [20]. In these situations, the JHDI can be used to triage the most vulnerable areas and create targeted campaigns to counter viral disinformation and encourage vaccination across platforms to fight disinformation.

To our knowledge, the JHDI is the first of its kind. Other indices such as the Global Disinformation Index focuses on disinformation produced by national governments [21]. Another similar index was formulated by CDC’s SVI, which endeavors to identify communities most vulnerable to natural disasters by using the data from the 2020 U.S. Census of Population and Housing and ranking each U.S. county (N=65,081) by the 15 variables within the U.S. census, such as income per capita, education, and single parenting [15]. The rank score is totaled for each county, and the county with the lowest total rank when all 15 variables were combined is deemed most vulnerable. However, there are several limitations to the CDC’s SVI.

First, CDC’s SVI is primed for identifying primarily communities with social and logistical barriers to receiving aid, whereas the JHDI identifies susceptibility of communities to disinformation. Additionally, CDC’s SVI gave equal weight to all 15 factors when ranking vulnerability. However, as we have shown with our index validation, applying significance factors can have a marked impact on index performance and provide more accurate data (Figures 2-4). Finally, the CDC’s SVI has not been validated against a national database for disinformation, whereas the JHDI was tested against the Yale Climate Change data with moderate correlation (R=0.4).

Limitations and next steps

The sourced data is fairly robust and reliable, and provides sufficient granularity for our purposes. However, there are several nuances that complicate this analysis, for example, misinformation on public health issues could be driven by microcultural circumstances that are not relevant to misinformation on other issues such as climate change. One documented example of this is the anti-vaccination tendencies among Somali communities; these populations may not have as strong opinions towards other issues such as global warming but feel strongly against anti-vaccination, which makes finding an association more difficult [5]. However, the rationale for exploring microcultural associations is strong with associations influenced by confounding factors. These associations can be explored in future studies with pattern analysis of social media platforms to find overlaps of association.

Furthermore, it would be interesting to understand the impact of local business patterns on public health misinformation. Misinformation has been used to protest lockdown measures in places where local economies have been hit the hardest; therefore, it might be interesting to investigate local economies. Starting with industries most impacted by the pandemic, counties that rely on them most can be selected for closer review. For example, it has been reported that meatpacking firms are facing investigation due to cases that have originated at packing locations [6]. It would be interesting to examine the nature of COVID-19 information dissemination in regions that rely on meatpacking for economic stability.

Additionally, variables such as lack of internet access, property crime rates, and population that are politically conservative are highly correlated with disinformation but were not included in this editions of the index due to incomplete county-level data [13]. Future studies can include these variables to the index to potentially augment disinformation susceptibility predictions. Furthermore, other databases such as non-medical vaccination exemption, mask-wearing belief, and Holocaust denial can also be used for validation of the index. However, caution should be used as the disinformation variables these databases contain may be influenced by additional social and political factors and are often less substantiated than the data from the Yale Climate Survey, resulting in a less accurate measure of disinformation.

Finally, it would be desirable to analyze a more dynamic data set. Currently, our data is relatively static; it captures snapshots of communities at specific points in accordance with census data. Moving forward, the model could be made more dynamic by incorporating news and social media sentiment analysis with advances in artificial intelligence. Doing so will make the JHDI an even more powerful tool in addressing disinformation.

## Conclusions

The JHDI has applications beyond improving public health outcomes around COVID-19. In an unprecedented era of mass communication, communities are at risk of losing empiricism and factfulness due to the mechanisms of sharing on social media. It is clear that as society evolves to incorporate new forms of media, institutions should evolve to adapt to new information ecosystems to promote fact-based information over mistruth. Using this index, local governments can now get a sense of how pervasive the problem of disinformation is and work to address disinformation proactively by using policy regulating social media, “info-interventions” to serve low-information populations with facts, and new civic media platforms designed with this problem in mind.

However, as with many types of repair work, the first step is figuring out how to measure where reality is broken.
